# 3D‐Printed Screw‐Rod Auxiliary System for Unstable Atlas Fractures: A Retrospective Analysis

**DOI:** 10.1111/os.13015

**Published:** 2021-04-07

**Authors:** Chao Wu, Jia‐yan Deng, Tao Li, Bo‐fang Zeng, Hai‐gang Hu, Yuan‐fang Zhu, Qin Wei

**Affiliations:** ^1^ Orthopaedics Center, Zigong Fourth People's Hospital Zigong China; ^2^ Digital Medical Center, Zigong Fourth People's Hospital Zigong China; ^3^ Health Management Center, Zigong Fourth People's Hospital Zigong China

**Keywords:** 3D printing, Atlas fractures, Navigation templates, Occipitocervical fusion, Occipitocervical inclination angle

## Abstract

**Objective:**

To develop and validate the efficacy of a 3D‐printed screw‐rod auxiliary system for unstable atlas fractures.

**Methods:**

This research is a retrospective analysis, and a total of 14 patients, including 11 males and three females, were enrolled in our hospital from January 2017 to March 2019 who underwent occipitocervical fusion assisted by the 3D‐printed screw‐rod auxiliary system were reviewed, and with an average age of 53.21 ± 14.81 years, an average body mass index (BMI) of 23.61 ± 1.93 kg/m^2^. The operation time, blood loss and radiation times during the operation were recorded. The maximum fracture displacement values of pre‐ and post‐operation were measured based on CT imaging. All screw grades were evaluated after surgery. The occipital‐cervical 2 (O‐C_2_) angle and occipitocervical inclination (OCI) angle of pre‐operation, post‐operation and the last following‐up were measured. The dysphagia scale 3 months after surgery and at the last follow‐up, the Neck Disability Index (NDI) 3 months after surgery and at the last follow‐up were assessed.

**Results:**

All patients were completed the surgery successfully. There was no patient with severe dysphagia or aggravation of nerve injury. The follow‐up was from 12 to 14 months, and with an average of 12.5 months. The average surgery time, average blood loss and average radiation times for the 14 patients were 112.14 min, 171.43 mL and 5.07 times, respectively. There was a significant difference in maximum fracture displacement between pre‐ and post‐operation values (*P* < 0.05). A total of 56 screws were inserted in 14 patients, among them, three screws were classified as grade 1, and the other screws were classified as grade 0. There was a significant difference in the O‐C_2_ between pre‐operation and 3 days after operation (*P* = 0.002); There was a significant difference in OCI angles between pre‐operation and 3 days after operation (*P* < 0.05); there was no significant difference in the O‐C_2_ or OCI angle between 3 days after the operation and the last follow‐up (*P* = 0.079; *P* = 0.201). The dysphagia scales of two patients were assessed as mild at 3 months after surgery, and the others were assessed as normal at 3 months after surgery. All patients' dysphagia scores returned to normal at the last follow‐up. The average NDI and average neck Visual Analogue Scale (VAS) scores at the last follow‐up were 2.53 and 8.41, respectively.

**Conclusion:**

It can objectively restore the OCI to normal with few post‐operative complications under the assistance of a screw‐rod auxiliary system to perform occipitocervical fusion for unstable atlas fractures and atlantooccipital joint instability.

## Graphical abstract

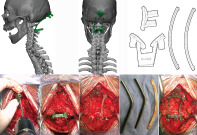

a, b. The screw‐rod auxiliary system was designed based on the virtual model and screws, lateral view and dorsal view; c. Structural drawing of the separated screw‐rod auxiliary system, including the reference model of the occipitocervical angle and the navigation templates of occipital screws and axial pedicle screws. d, Occipital screws were inserted, assisted by the navigation template; e. Axial pedicle screws were inserted, assisted by the navigation template; f. The occipitocervical angle was adjusted according to the reference model of the occipitocervical angle; g. According to the occipitocervical angle reference model, the connecting rod with an appropriate length was applied and then bent; h. Completed occipitocervical fusion.

## Introduction

Fractures of the atlas account for 3%–13% of cervical spine fractures^1^. There is still much controversy about the treatment of C_1_ fractures^2^. The most common fracture classification is the Gehweiler classification: type 1 fractures are isolated fractures of the anterior ring of the atlas, type 2 refers to isolated fractures of the posterior arch of the atlas, type 3 are combined fractures of the anterior and posterior arches of the atlas, type 4 refers to isolated fractures of the lateral mass, and type 5 refers to fractures of the transverse process^3^, ^4^. Types 3 and 4 are considered unstable fractures and often require surgery^4^. The surgical methods include anterior atlantoaxial fusion^5^, posterior atlantoaxial transarticular screw fixation^6^, posterior atlantoaxial pedicle screw fixation^7^, etc. Occipital‐cervical 2 (O‐C_2_) fusion is required in patients with unstable atlas fractures complicated by atlantooccipital instability and difficulty placing lateral mass screws or pedicle screws^8^, ^9^. In this surgery, the placement of axial screws and occipital screws accurately and safely, as well as the return to normal of the occipitocervical inclination (OCI), is particularly important^10^. There is a high failure rate of screw placement based on anatomical markers^11^. Complications such as dysphagia and dyspnoea may occur when the cervical occiput is fused at an abnormal angle. Yoshid *et al*. first reported a patient with rheumatoid arthritis who had developed serious dyspnoea and dysphagia immediately after a short occipitocervical fusion^12^, which resulted in a failure of occipitocervical fixation.

With the clinical application of various navigation technologies, the success rate of such operations obtained an improvement. Wang *et al*. ^13^ performed occipitocervical fusion on eight patients under o‐arm navigation, and all the patients exhibited radiographic evidence of osseous fusion by X‐ray and CT, and no neurovascular complications occurred. Thirty‐two of the 34 screws were rated grade 1, and the other two screws broke through the pedicle wall. The safety of screw placement was as high as 94%. Fiorenza and Ascanio^14^ performed posterior upper cervical fixation on 21 patients under 3D navigation system with intraoperative single level vertebral registration on pre‐operative cervical CT/CTA; From the post‐operative CT, all screws position appeared satisfactory, and no neurovascular damage occurred in all patients. Although the above techniques have improved the safety of pedicle screw placement to a certain extent, there are also cases that screws break through the pedicle, and these techniques also increase the radiation for patients.

However, with the development of 3D printing technology, the above problems have been mostly resolved. Yuan *et al*. ^15^ used a 3D navigation system and 3D printing technology in a patient diagnosed with atlantoaxial dislocation and basilar invagination. The patient's walking disorder was resolved and he was able to walk approximately 100 m by himself with the help of a neck brace after surgery. At 6 months after surgery, the patient reported that the numbness of the limbs was reduced, and he could walk more than 500 m by himself. The most important advantage of 3D printing technology in surgery in that it can clarify the relationship between blood vessels and bone around the implant to minimize injury to important structures during implantation. Ganesha *et al*.^16^ made a patient‐specific 3D‐printed implant and tool for occipitocervical fixation, which used for operative planning, patient education, and intraoperative reference, and the patient‐specific implant was pre‐contoured to match the posterior occipitocervical bony spine and reproduce the planned occipitocervical “neutral” position, and with no intraoperative or post‐operative complications occurred. The patient reported resolution of symptoms and demonstrated satisfactory occipitocervical alignment without evidence of implant dysfunction at 6‐month follow‐up. All the above studies reflect the significant advantages of 3D printing technology in occipitocervical fusion surgery. However, they are case reports, and we hope to confirm and summarize their advantages through more case studies.

In this study, we used 3D printing technology to develop a set of patient‐specific navigation templates for the placement of axial pedicle screws and occipital screws and a reference model of the OCI for restoring the normal OCI to improve the screw placement accuracy and reduce the complications caused by an inappropriate OCI. To our knowledge, this is the first report of OCI recovery using a patient‐specific reference model of the OCI.

The aims of this study are as follows: (i) during surgery, the surgical assistant kept detailed records of operation time, bleeding and transmission fluoroscopy; The above intraoperative indexes was used to explore the feasibility of the patient‐specific screw‐rod auxiliary system for assisting with occipitocervical fixation in unstable atlas fractures and atlantooccipital joint instability: (ii) the grading of screws, O‐C_2_ angle and OCI were measured based on the radiographic images to research the insertion accuracy of the axis and occipital screws assisted by the navigation template; and (iii) the Dysphagia scale, Neck Disability Index (NDI) and neck Visual Analogue Scale (VAS) after surgery were evaluated to investigate the effectiveness of the patient‐specific reference model of the OCI.

## Materials and Methods

This study was approved by Zigong No. 4 People's Hospital Review Board (IRB Number, 2017‐002). From January 2017 to March 2019, a total of 14 patients were enrolled in our research, 11 males and three females, with an average age of 53.21 years and an average body mass index (BMI) of 23.61 kg/m^2^. Six of them were classified as type 3 fractures, and eight patients were classified as type 4 fractures according to the Gehweiler classification. Seven patients showed atlantooccipital joint instability, and 10 patients experienced screw placement difficulty. One patient was diagnosed with ASIA grade D, and the others were ASIA grade E (Table 1). The operations were performed by the same surgeon with more than 10 years experience, and all patients were informed of the experimental design before the surgery and signed an informed consent form.

**TABLE 1:** General information of patients treated by reference model and navigation templates

No.	Gender	Age (years)	Body mass index (kg/m^2^)	Gehweiler classification	Atlantooccipital joint instability	Screw placement difficulty	ASIA grade
P1	Female	58	24.6	Type 3	Y	Y	E
P2	Female	44	26.2	Type 4	Y	Y	E
P3	Male	81	21.7	Type 4	N	Y	E
P4	Male	49	22.8	Type 4	N	Y	D
P5	Male	71	21.5	Type 3	Y	N	E
P6	Male	75	25.5	Type 3	Y	Y	E
P7	Female	48	23.7	Type 4	N	Y	E
P8	Male	54	21.9	Type 4	N	Y	E
P9	Male	42	23.8	Type 4	N	Y	E
P10	Male	57	22.6	Type 4	N	Y	E
P11	Male	56	24.6	Type 3	Y	N	E
P12	Male	33	22.3	Type 3	Y	N	E
P13	Male	30	27.8	Type 3	Y	N	E
P14	Male	47	21.6	Type 4	N	Y	E

The inclusion criteria were as follows: (i) atlas fractures; Gehweiler classification types 3 and 4 combined with atlantooccipital instability; screw placement difficulty; (ii) aged 30 to 81 years; (iii) maximum displacement of the fracture, O‐C_2_ angle, and OCI were compared; and (iv) followed for more than 1 year. The exclusion criteria were as follows: (i) patients with pathological fractures; (ii) severe systemic diseases; and (iii) severe osteoporosis.

### Model Construction of the Cervical Occiput

**Fig. 1:**
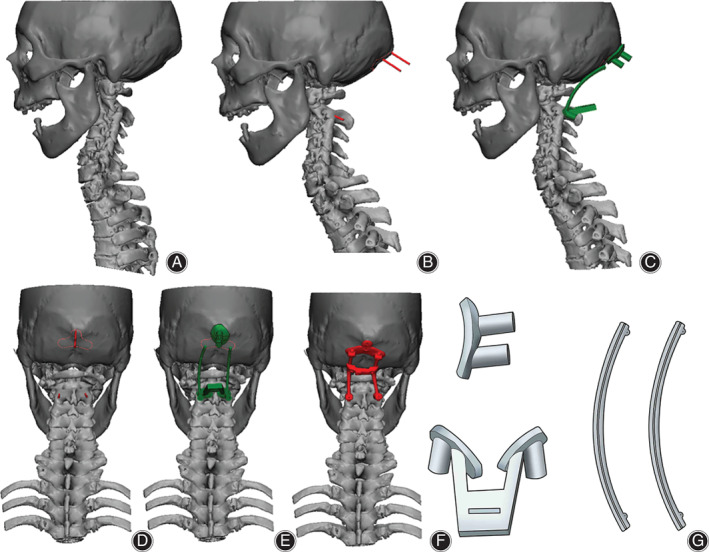
Model establishment and design of the screw‐rod auxiliary system. (A) Constructed model of the cervical occiput, lateral view and dorsal view; (B, D) the virtual occipital screws and axial pedicle screws were placed, lateral view and dorsal view; (C, E) the screw‐rod auxiliary system was designed based on the virtual model and screws, lateral view and dorsal view; (F) the screw‐rod auxiliary system was fixed; (G) structural drawing of the separated screw‐rod auxiliary system, including the reference model of the occipitocervical angle and the navigation templates of occipital screws and axial pedicle screws.

CT of the cervical occiput was obtained in DICOM format and then imported into Mimics 21.0 (Materialise, Leuven, Belgium). The CT value of bone was selected as the threshold to extract the mask, and then the 3D model of the cervical occiput was calculated based on the mask (Fig. 1A, D). The virtual screws were placed in the pedicle of the axis, and virtual screws of the occiput were placed in the occipital tuberosity based on the 3D models. According to the measurement and conclusion of Shoda *et al*. ^17^, in the sagittal plane, the occiput was rotated to maintain an O‐C_2_ angle of 14° (Fig. 1B, E). The left and right splines were drawn along the occipital screws, atlas, and axis pedicle screws.

### Design of Screw‐rod Auxiliary System

The splines and virtual screws described above were imported into 3‐matic (Materialise, Leuven, Belgium). The spline diameter was set to 3.2 mm as the reference model, and both terminals of each reference model were designed with a point to mark the position of the screw on the cervical occipital surface. The virtual screw diameter was set to 2.1 mm. The bone surface structure around the screw insert point was extracted and stretched to a thickness of 3 mm to serve as the basis of the navigation templates. A hollow pipe with an inner diameter of 2.1 mm and a length of 2 cm along the direction of the virtual screw was designed as the guide pipe (Fig. 1C, F). Then, the base and guide pipes were united as the navigation template. The reference model and navigation template were collectively referred to as the screw‐rod auxiliary system (Fig. 1G).

### Preoperative Preparation

**Fig. 2:**
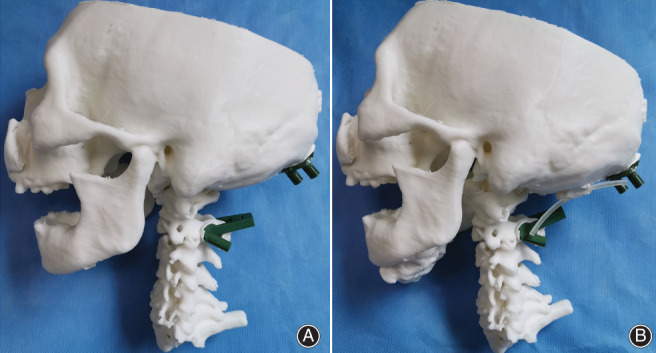
Simulated surgery based on the 3D‐printed model. (A) Simulated screw placement assisted by the 3D‐printed navigation template; (B) the reference model of the occipitocervical angle was placed to verify its effectiveness.

Including standard anteroposterior and lateral radiographs, CT and MRI were performed to assess the fracture type and the integrity of the intervertebral discs and ligamentous injuries. The pre‐operative operation was simulated on the 3D‐printed model. The axial and occipital navigation templates were matched with the corresponding positions of the model. A diameter of 2 mm for the K‐wires was drilled through the guide pipes, and the route of the K‐wires was observed to verify navigation template accuracy (Fig. 2A). The reference model of the OCI was assembled to verify sagittal curvature correction (Fig. 2B). The screw‐rod auxiliary system was sterilized at low temperature before the operation.

### Surgical Technique

#### Anesthesia and Position

Under general anesthesia, the prone position was recommended during the surgical procedure on a radiolucent operation table with the head fixed in a Mayfield head holder.

#### Approach and Exposure

*Via* a posterior median approach, surgical exposure was accomplished from the occiput to the C_2_ spinous process. The muscle and subcutaneous fascia tissues were fully stripped from the bones.

#### Fixation or Placement of the Prosthesis

**Fig. 3:**
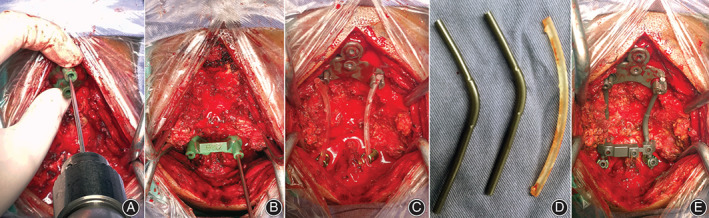
Surgical procedure. (A) Occipital screws were inserted, assisted by the navigation template; (B) axial pedicle screws were inserted, assisted by the navigation template; (C) the occipitocervical angle was adjusted according to the reference model of the occipitocervical angle; (D) according to the occipitocervical angle reference model, the connecting rod with an appropriate length was applied and then bent; (E) completed occipitocervical fusion.

Occipital screw navigation templates and axial screw navigation templates were placed in the back of the corresponding bone and fixed firmly by the assistant; two K‐wires were drilled step by step into the axial pedicle through the axial guide pipes. The pedicle screws were placed after the channel was detected (Fig. 3A); two K‐wires were drilled into the occiput through the occipital guide holes (Fig. 3B). An occipital screw‐plate was fixed to the keel of the sub‐occipital cranium (Fig. 3C).

#### Reconstruction

Two connecting rods were contoured to refer to the shape and length of the patient‐specific reference model (Fig. 3D). The next step was to firmly fix the locking caps of the C_2_ pedicle screw and to place the locking caps loosely on the occipital screw plate so that the rod was firmly attached to the C_2_ pedicle screw while sliding freely within the occipital screw plate. According to the shape of the reference model, the O‐C_2_ angle was adjusted; according to the distance of the dots on the reference model, the occipital locking caps were tightened firmly while maintaining distraction (Fig. 3C). Finally, all locking caps were securely tightened (Fig. 3E). After satisfactory reduction, autologous bone grafts from the ilium were obtained for bone arthrodesis and stratified suturing of the wound.

### General Indicator

#### Operative Time

In our study, the operation time was defined as the period between wound opening and wound closing. Less operation time can effectively reduce the operation risk.

#### Blood Loss

The amount of blood loss during the operation can reflect the quality of the operation.

Less blood loss can accelerated rehabilitation for patients.

#### Radiation Times

The use of c‐arm fluoroscopy during the whole operation, fewer radiation times can reduce the radiation exposure to operators and patients.

### Radiographic Indicator

#### Maximum Displacement of the Fracture

The maximum displacement of the fracture end of the fracture was measured based on CT imaging, and the maximum displacement reflect the fracture reduction.

#### O‐C_2_ Angle

**Fig. 4:**
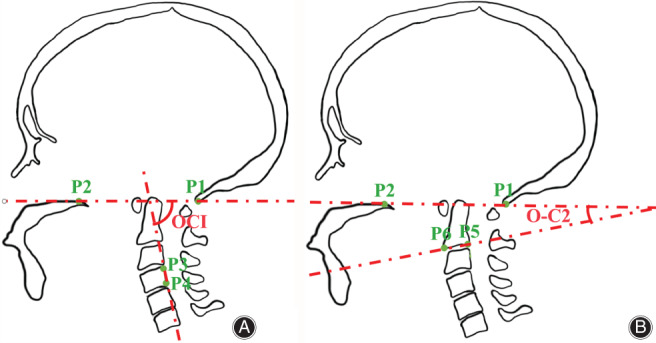
(A) Measurement of the OCI angle. Point 1 is the most caudal point on the midline occipital curve, point 2 is located in the posterosuperior aspect of the hard palate; Point 3 and point 4 are located in the vertebral body edge of C_4_. The OCI angle is defined as the angle between McGregor's line and the vertebral body edge of C_4_. (B) Measurement of the O‐C_2_ angle. Points 5 and 6 are located in the inferior endplate of the axis. The O‐C_2_ angle is defined as the angle between McGregor's line and the inferior endplate line of C_2_.

The angle between the McGregor's line and the inferior endplate line of C_2_ was measured as the O‐C_2_ angle (Fig. 4A). A normal person's O‐C_2_ Angle is close to 14°, and an abnormal O‐C_2_ angles may cause difficulty in swallowing or dyspnoea.

#### OCI

the posterior border of C_4_ was measured as the OCI angle ^17^ (Fig. 4B), and abnormal OCI angles may cause difficulty in swallowing or dyspnoea.

### Clinical Indicator

#### Dysphagia Scale

The dysphagia scale was evaluated as follows: normal refers to patients without any swallowing difficulties; mild refers to patients with rare, intermittent episodes of dysphagia; moderate refers to patients with some difficulty when swallowing some special food; and severe refers to patients with difficulty even swallowing liquid^18^.

#### NDI

**Fig. 5:**
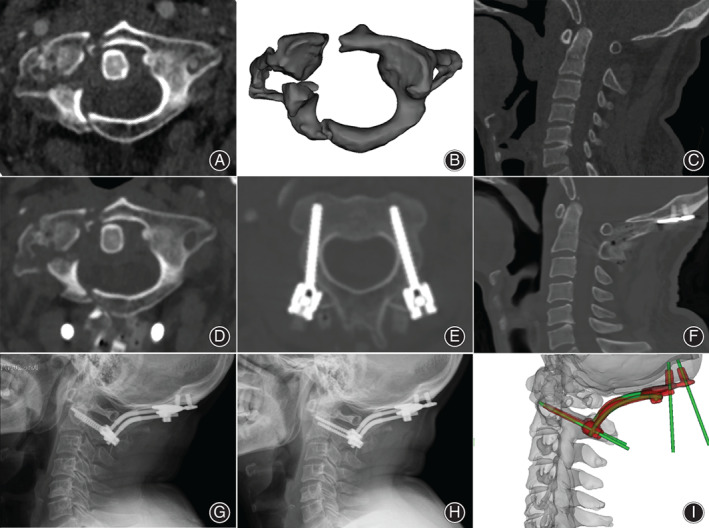
The imaging of patient NO. 10, male, diagnosed with a fracture of the lateral mass of C_1_, type 4. (A) Axial CT of C_2_; (B) 3D imaging of C_2_; (C) Pre‐operation O‐C_2_ angle of 6.71°; (D) postoperative atlas reduction; (E) C_2_ pedicle screw with grade 0; (F) occipital screw with grade 0; (G) OCI angle of 79.56° and O‐C_2_ angle of 14.12° immediately post‐operation; (H) OCI angle of 77.45° and O‐C_2_ angle of 14.79° at the last follow‐up; (I) registration of post‐ and pre‐operation CT, screw placement and occipitocervical angle consistent with the preoperative design.

NDI was used to evaluate neck pain and disability ^19^. NDI contains 10 self‐reported items, including: pain intensity, personal care, lifting, reading, headache, concentration, working, sleeping, driving, and entertainment. Each item is scored from zero to five. The final score was presented as the percentage of the maximal score. Final NDI score is calculated as (total score/(five × number of questions answered)) × 100%. 0% to 20% is considered mild dysfunction, 21%–40% is moderate dysfunction, 41%–60% is severe dysfunction, and 61%–80% is considered as disability. 81 percent to 100% is either long‐term bedridden or exaggerating the impact of pain on their life (Fig. 5).

#### VAS

The VAS score system used in the social and behavioral sciences to measure low back pain and leg pain ^20^. The VAS pain scoring standard (scores from 0 to 10) was as following: 0 means painless; 1–3 means mild pain that the patient could endure; 4–6 means patient was in pain that could be endured and be able to sleep; and 7–10 means patient had intense pain and was unable to tolerate the pain.

### Screw Grading

The grading score was used to evaluate the safety of the pedicle screw^21^; grade 0: no breach; grade 1: breach less than 2 mm; grade 2: breach of 2 to 4 mm; and grade 3: breach greater than 4 mm. Grade 0 and grade 1 placements were considered successful and safe, and grade 2 and grade 3 placement indicated the possibility of nerve damage.

### Statistical Analysis

All statistical analyses were performed in SPSS 19.0 (SPSS Inc.; Chicago, IL, USA). Descriptive statistics were performed for a single set of quantitative data, including the mean value, standard deviation, minimum value and maximum value, and for age, BMI, surgical time, blood loss, radiation times, NDI and neck VAS. The paired *t*‐test was performed for pre‐operative and post‐operative comparison of quantitative data, including the maximum displacement of the fracture between pre‐operation and post‐operation values. A one‐way repeated measure (ANOVA) was performed for multiple sets of comparative quantitative data, including the O‐C_2_ angle and OCI angle pre‐operation, 3 days after the operation and at the last follow‐up. The level of statistical significance was set at 0.05 (two‐tailed).

## Results

### Follow‐up

All patients were followed up for 12 to 14 months. CT, X‐ray and clinical examinations were performed in all patients within 3 days after surgery and at the last follow‐up. The grading of screws, O‐C_2_ Angle and OCI of 3 days after surgery and the last following‐up were measured based on the radiation imaging. The Dysphagia Scale, NDI and Neck VAS within 3 days after surgery and the last following‐up were evaluated.

### General Indicator

The average surgical time, blood loss and radiation times were 112.14 ± 15.78 (range, 90 to 140) min, 171.43 ± 73.05 (range, 80 to 300) mL and 5.07 ± 1 times (range, 4 to 6), respectively.

### Radiographic Improvement

The maximum displacement of the fracture was 6.95 ± 3.19 mm before surgery and 4.75 ± 2.57 mm within 3 days after surgery, and there was a significant difference between pre‐ and post‐operation values (*t* = 5.40, *P* = 0.000). There was a significant difference in the O‐C_2_ angle (*t* = 4.51, *P* = 0.002) and OCI angle (*t* = 8.11, *P* = 0.000) between values pre‐operation and those 3 days after the operation. There was no significant difference compared O‐C_2_ angle with 14° (*t* = 0.016, *P* = 0.99). There was no significant difference in the O‐C_2_ angle (*t* = 2.23, *P* = 0.079) or OCI angle (*t* = 1.56, *P* = 0.201) between values 3 days after the operation and those at the last follow‐up.

### Clinical Improvement

One patient with ASIA from grade D recovered to ASIA grade E at the last follow‐up. The dysphagia scale was assessed as “mild” in two patients, and in the other patients, the dysphagia scale was assessed as “normal” 3 months after the operation. All patients had “normal” dysphagia scales 12 months after the operation. The average NDI and neck VAS scores 12 months after the operation were 2.53 ± 0.4 (range, 2.07 to 3.16) and 8.41 ± 0.75 (range, 7.14 to 9.75), respectively (Table 2).

**TABLE 2:** Clinical and radiological results of patients

No.	Surgical time(min)	Blood loss(mL)	Radiation times m	Maximum displacement of fracture, Preop (mm)	Maximum displacement of fracture, Post‐3d (mm)	Grade of C2		Grade of occipital		O‐C2 angle			OCI angle			Dysphagia scale, postop 3m	Dysphagia scale, Post‐last	NDI, Post‐last	Neck VAS, Post‐last
						L	R	O1	O2	Pre‐S	Post‐3d	Post‐last	Pre‐S	Post‐3d	Post‐last				
P1	100	200	4	6.21	3.23	0	0	0	0	7.56	14.23	15.17	103.23	78.23	76.46	Mild	Normal	3.12	8.52
P2	110	250	6	7.78	4.14	1	0	0	1	8.56	14.54	15.10	102.52	88.96	86.59	Normal	Normal	2.07	9.25
P3	110	150	4	9.37	5.50	0	0	0	0	9.61	15.13	14.52	101.51	84.84	82.45	Normal	Normal	3.16	7.56
P4	90	100	4	12.23	7.81	0	0	0	0	11.73	12.56	13.56	99.09	87.63	85.46	Normal	Normal	2.08	8.52
P5	120	80	6	7.84	5.5	0	0	0	0	6.06	14.35	14.12	112.74	78.52	76.23	Normal	Normal	2.12	8.48
P6	140	300	5	13.42	11.51	0	0	0	0	10.13	15.32	14.28	106.92	86.54	84.78	Normal	Normal	2.56	8.56
P7	100	150	4	3.54	2.13	0	0	0	0	9.85	14.12	14.86	101.88	89.41	87.45	Mild	Normal	2.45	8.12
P8	120	200	6	2.85	1.24	0	0	0	0	11.14	14.82	15.55	105.42	81.13	79.87	Normal	Normal	2.14	8.56
P9	110	100	6	2.98	4.34	0	0	0	0	15.23	12.23	13.25	98.56	92.36	99.47	Normal	Normal	3.15	8.12
P10	90	100	4	7.25	4.26	0	0	0	0	6.71	14.12	14.79	115.27	79.56	77.45	Normal	Normal	2.48	8.17
P11	120	150	6	5.23	2.25	0	0	0	0	8.17	12.34	13.86	107.29	85.54	83.42	Normal	Normal	2.49	9.75
P12	120	120	6	4.23	4.10	0	1	0	0	12.89	13.44	14.10	104.23	86.45	84.47	Normal	Normal	2.69	7.48
P13	140	300	6	7.56	5.36	0	0	0	0	14.83	14.78	14.98	89.19	80.56	78.89	Normal	Normal	2.16	9.56
P14	100	200	4	6.82	5.23	0	0	0	0	9.63	13.96	14.25	96.53	79.31	77.78	Normal	Normal	2.78	7.14
Mean	112.14	171.43	5.07	6.95	4.75					10.15	14.00	14.46	103.17	84.22	82.91			2.53	8.41
SD	15.78	73.05	1.00	3.19	2.57					2.90	1.04	0.68	6.52	4.57	6.20			0.4	0.75
Statistic				T = 5.4						*P* = 0.002*			*P* = 0.000*						
				*P* = 0.000							*P* = 0.079^†^			*P* = 0.201^†^					

* , Comparison between pre‐operation *VS* 3 days after operation,

† , Comparison between 3 days after operation *vs* last following‐up.

### Screw Grading

A total of 28 C_2_ screws and 28 occipital screws were placed. One left C_2_ screw, one right C_2_ screw and ine occipital screw had grade 1 placement, and the other screws had grade 0 placement.

### Complications

None of the patients had complications such as infection, severe dysphagia or nerve damage after surgery.

## Discussion

### Feasibility of the Patient‐specific Screw‐rod Auxiliary System

Miyata *et al*. analyzed the O‐C_2_ angle in 29 patients who underwent occipitocervical fusion and found that the O‐C_2_ angle had a significant impact on post‐operative dyspnoea and/or dysphagia^22^. Yoon suggested that the OCI angle is an important parameter reflecting the OCI in patients with anatomic abnormalities of C_0_‐C_2_ ^23^. Shoda *et al*. put forth that the McGregor line is the most reproducible and reliable method for measurement of the OCI angle^17^; Nagashima *et al*. suggested the use of the Oc‐Ax angle instead of the O‐C_2_ angle when it was difficult to find the McGregor line or endplate of C_2_ ^24^. The O‐C_2_ and OCI angles have been used as important parameters to evaluate OCI recovery because they are easy to measure during surgery^22^. Sitoula *et al*. recommended a technology for occipitocervical fusion, and it helps restore the OCI and maintains screw stabilization^25^. During the operation, C‐arm fluoroscopy has recently been used to assess the recovery of the OCI; however, this method has substantial subjectivity and needs multiple perspectives to adjust the angle. In this study, we used a patient‐specific 3D‐printed screw‐rod auxiliary system to assist with screw placement and pre‐bending of the rod to improve the objectivity of intraoperative confirmation and improve the reduction effect.

### Effectiveness of the Screw‐rod Auxiliary System

In this study, we achieved a great therapeutic effect with the screw‐rod auxiliary system. First, the average operation time of the 14 patients was 112.14 min, and the average blood loss was 171.43 mL in this study, which was significantly less than that with this type of surgery among recent studies ^26^, ^27^. The average radiation times in the study was 5.07, which was significantly less than that in previous studies ^16^, ^28^. Second, the degree of pos‐toperative fracture displacement was significantly less than that of pre‐operative fracture displacement, indicating that this technique could effectively reduce the fracture. Third, this technology to assist screw placement is safe, and no screws more severe than grade 1 were found in this study. Fourth, the OCI was well restored to be consistent with the pre‐operative design and with an average O‐C_2_ angle of 14° after surgery. The fixation was stable, since there was no significant difference in the O‐C_2_ or OCI angle between the values post‐operation and at the last follow‐up. Last, dyspnoea was rarely seen in all 14 patients, and the ASIA scale fully recovered to grade E, with satisfactory NDI and VAS scores at the last post‐operative follow‐up.

### Operation Skills

Here, we provide some tips for this type of surgery; (i) the simulated reduction of the fracture site should be performed before surgery, and the OCI should be adjusted from the sagittal position and coronal position to satisfy physiological curvature; (ii) the base of the axial navigation template should cover the root of the axial spinous process, and the base of the occipital navigation template should cover the occipital nodule to ensure that the navigation template is firmly attached to the bone and to improve the accuracy of screw placement; (iii) the inner diameter of the guide pipe is reserved with a mobility of 0.1 mm for the passage of the K‐wires. The length of the guide pipe should be designed to be 15–20 mm to accurately guide while avoiding excessive soft tissue stripping; (iv) it is best to choose a K‐wire with a limited depth and a diameter of 2 mm, and the K‐wires should be deepened step by step to explore the screw corridor; (v) during the operation, the soft tissue around the bone attached by the base of the navigation template should be completely removed to ensure firm attachment of the navigation template; (vi) in order to better expose the occiput and the C_2_ spinous process, we usually reduce the O‐C_2_ angle byadjusting the Mayfield head holder in the pre‐operative. If we find the actual O‐C_2_ angle is much different from the pre‐operative design during surgery, we usually release the rotating head device of Mayfield head holder by the assistant to change the O‐C_2_ angle.In this way, the O‐C_2_ angle can be restored objectively according to the 3D‐printed screw‐rod auxiliary system; and(vii) after O‐C_2_ angle fixation, the screw position and recovery of the O‐C_2_ angle should be reconfirmed by C‐arm fluoroscopy.

### Limitations

There are some limitations of this research that should be noted. First, we measured the O‐C_2_ angle and OCI angle in this study, but these two indicators were not sufficient to evaluate the recovery of the OCI. Second, some complications of the patients may have been ignored, and patients' post‐operative statuses could not be fully evaluated due to the short follow‐up. Finally, only 14 patients were enrolled in this study, resulting in a relatively small sample size. In a later study, we will add the anatomical measurement index and clinical observation index, prolong the follow‐up and expand the sample size.

### Conclusion

It is clinically feasible with the assistance of a screw‐rod auxiliary system to perform occipitocervical fusion for unstable atlas fractures. This novel technique can objectively restore the angle OCI of patients, and there are few postoperative complications.

## Declarations

This study was approved by the ethics committee of Zigong Fourth People's Hospital (No. 02, 2013). All patients signed the informed consents to participate in the study.

## Disclosure

No benefits in any form have been or will be received from a commercial party related directly or indirectly to the subject of this manuscript. There is no conflict of interest between authors.

## Funding

This study was supported by Sichuan Key Science and Technology Plan Project (2016JY0108) and Zigong key science and technology project (2019YLSF05).

## References

[1] Kim HS , Cloney MB , Koski TR , Smith ZA , Dahdaleh NS . Management of isolated atlas fractures: a retrospective study of 65 patients. World Neurosurg, 2017, 111: 316–322. 10.1016/j.wneu.2017.12.053 29258944

[2] Vieweg U , Meyer B , Schramm J . Differential treatment in acute upper cervical spine injuries. Surg Neurol, 2000, 54: 203–210. 11118566 10.1016/s0090-3019(00)00301-3

[3] Harms J , Melcher RP . Posterior C1‐C2 fusion with polyaxial screw and rod fixation. Spine (Phila Pa 1976), 2001, 26: 2467–2471. 11707712 10.1097/00007632-200111150-00014

[4] Kim MK , Shin JJ . Comparison of radiological and clinical outcomes after surgical reduction with fixation or halo‐vest immobilization for treating unstable atlas fractures. Acta Neurochir, 2019, 161: 685–693. 30710241 10.1007/s00701-019-03824-5

[5] Ostolides PJ , Theodore N , Karahalios DG , Sonntag VK . Triple anterior screw fixation of all acute combination arias—axis fracture. Case Report J Neurosurg, 1997, 87: 96–99. 9202272 10.3171/jns.1997.87.1.0096

[6] Richter M , Schmidi R , Claes L , Puhl W , Wilke HJ . Posterior atlantoaxial fixation: biomechanical in vitro comparison of six different techniques. Spine (Phila Pa 1976), 2002, 27: 1724–1732. 12195062 10.1097/00007632-200208150-00008

[7] Resniek DK , Benzel EC . Cl‐C2 pedicle screw fixation with rigid cantilever bean construct: case report and technical note. Neurosurgery, 2002, 51: 853–854. 12229870

[8] Guigui P , Milaire M , Morvan G , Lassale B , Deburge A . Traumatic atlantooccipital dislocation with survival: case report and review of the literature. Eur Spine J, 1995, 4: 242–247. 8528784 10.1007/BF00303419

[9] Labler L , Eid K , Platz A , Trentz O , Kossmann T . Atlanto‐occipital dislocation: four case reports of survival in adults and review of the literature. Eur Spine J, 2004, 13: 172–180. 14673716 10.1007/s00586-003-0653-5 PMC3476575

[10] Tang C , Li GZ , Liao YH , Tang Q , Ma F , Wang Q . Importance of the occipitoaxial angle and posterior occipitocervical angle in occipitocervical fusion. Orthop Surg, 2019, 11: 1054–1063. 31743954 10.1111/os.12553 PMC6904633

[11] Diaz R , Berbeo M , Villalobos L , Vergara M . Minimally invasive posterior C1‐C2 screw fixation through an anatomical corridor preserving occipitocervical tension band: prospective 21 months clinical and radiological study. Spine J, 2009, 9: 24S.

[12] Yoshida M , Neo M , Fujibayashi S , Nakamura T . Upper‐airway obstruction after short posterior Occipitocervical fusion in a flexed position. Spine (Phila Pa 1976), 2007, 32: E267–E270. 17426623 10.1097/01.brs.0000259977.69726.6f

[13] Wang YC , Zhou ZZ , Wang B , *et al*. Occipitocervical fusion via cervical pedicle fixation assisted with O‐arm navigation. Orthop Surg, 2020, 12: 1100–1107. 32697041 10.1111/os.12704 PMC7454157

[14] Fiorenza V , Ascanio F . Safety and efficacy of posterior atlanto‐axial stabilization using intraoperative navigation system with preoperative computed tomographic scan. World Neurosurg, 2019, 129: 110–119. 31170507 10.1016/j.wneu.2019.05.242

[15] Yuan T , Jia G , Yang L , Xu D , Zhang J , Liu Q . Occipitocervical fusion combined with 3‐dimensional navigation and 3‐dimensional printing technology for the treatment of atlantoaxial dislocation with basilar invagination: a case report. Medicine, 2020, 99: e18983–e18988. 32000432 10.1097/MD.0000000000018983 PMC7004706

[16] Thayaparan GK , Owbridge MG , Thompson RG , D'Urso PS . Patient‐specific processes for occipitocervical fixation using biomodelling and additive manufacturing. J Clin Neurosci, 2020, 71: 251–256. 31677900 10.1016/j.jocn.2019.10.005

[17] Shoda N , Takeshita K , Seichi A , *et al*. Measurement of occipitocervical angle. Spine (Phila Pa 1976), 2004, 29: E204–E208. 15131455 10.1097/00007632-200405150-00022

[18] Wang X , Chou D , Jian F . Influence of postoperative O‐C2 angle on the development of dysphagia after occipitocervical fusion surgery: results from a retrospective analysis and prospective validation. World Neurosurg, 2018, 116: E595–E601. 29777893 10.1016/j.wneu.2018.05.047

[19] Han X , He D , Zhang N , Song Q , Wang J , Tian W . Comparison of 10‐year outcomes of Bryan cervical disc arthroplasty for myelopathy and radiculopathy. Orthop Surg, 2019, 11: 1127–1134. 31762194 10.1111/os.12565 PMC6904630

[20] Liang JQ , Chen C , Zhao H . Revision surgery after percutaneous endoscopic Transforaminal discectomy compared with primary open surgery for symptomatic lumbar degenerative disease. Orthop Surg, 2019, 11: 620–627. 31402585 10.1111/os.12507 PMC6712385

[21] Gertzbein SD , Robbins SE . Accuracy of pedicular screw placement in vivo. Spine (Phila Pa 1976), 1990, 15: 11–14. 2326693 10.1097/00007632-199001000-00004

[22] Miyata M , Neo M , Fujibayashi S , Ito H , Takemoto M , Nakamura T . O‐C2 angle as a predictor of dyspnea and/or dysphagia after occipitocervical fusion. Spine (Phila Pa 1976), 2009, 34: 184–188. 19139669 10.1097/BRS.0b013e31818ff64e

[23] Yoon SD , Lee CH , Lee J , Choi JY , Min WK . Occipitocervical inclination: new radiographic parameter of neutral occipitocervical position. Eur Spine J, 2017, 26: 2297–2302. 28555311 10.1007/s00586-017-5161-0

[24] Nagashima S , Nagae M , Arai Y , *et al*. A new method of measuring the occipitocervical angle that could be applied as an intraoperative indicator during occipitocervical fusion. Clin Spine Surg, 2016, 30: E981–E987. 10.1097/BSD.0000000000000478 27906740

[25] Sitoula P , Mackenzie WG , Shah SA , *et al*. Occipitocervical fusion in skeletal dysplasia: a new surgical technique. Spine (Phila Pa 1976), 2014, 39: E912–E918. 24825152 10.1097/BRS.0000000000000381

[26] Winegar CD , Lawrence JP , Friel BC , *et al*. A systematic review of occipital cervical fusion: techniques and outcomes. J Neurosurg Spine, 2010, 13: 5–16. 20594011 10.3171/2010.3.SPINE08143

[27] Ding X , Abumi K , Ito M , *et al*. A retrospective study of congenital osseous anomalies at the craniocervical junction treated by occipitocervical plate‐rod systems. Eur Spine J, 2012, 21: 1580–1589. 22547213 10.1007/s00586-012-2324-x PMC3535228

[28] Rajasekaran S , Soundararajan DCR , Shetty AP , Kanna RM . Motion‐preserving navigated primary internal fixation of unstable C1 fractures. Asian Spine J, 2020, 14: 466–474. 32050311 10.31616/asj.2019.0189 PMC7435319

